# Shifts in Developmental Timing, and Not Increased Levels of Experience-Dependent Neuronal Activity, Promote Barrel Expansion in the Primary Somatosensory Cortex of Rats Enucleated at Birth

**DOI:** 10.1371/journal.pone.0054940

**Published:** 2013-01-25

**Authors:** Ingrid Fetter-Pruneda, Helga Geovannini-Acuña, Cecilia Santiago, Ana Sofía Ibarrarán-Viniegra, Eduardo Martínez-Martínez, Marcela Sandoval-Velasco, Laura Uribe-Figueroa, Patricia Padilla-Cortés, Gabriela Mercado-Célis, Gabriel Gutiérrez-Ospina

**Affiliations:** 1 Departmento de Biología Celular y Fisiología, Programa de Posgrado en Ciencias Bilógicas, Instituto de Investigaciones Biomédicas, Universidad Nacional Autónoma de México, México, Distrito Federal, México; 2 Laboratorio de Oncogenómica, Dirección de Investigación Médica, Instituto Nacional de Medicina Genómica, México, Distrito Federal, México; 3 Unidad de Genotipificación y Análisis de Expresión Affymetrix, Instituto Nacional de Medicina Genómica, México, Distrito Federal, México; 4 Unidad de HPLC, Instituto de Investigaciones Biomédicas, Universidad Nacional Autónoma de México, México, Distrito Federal, México; Federal University of Rio de Janeiro, Brazil

## Abstract

Birth-enucleated rodents display enlarged representations of whiskers (i.e., barrels of the posteromedial subfield) in the primary somatosensory cortex. Although the historical view maintains that barrel expansion is due to incremental increases in neuronal activity along the trigeminal pathway during postnatal development, recent evidence obtained in experimental models of intramodal plasticity challenges this view. Here, we re-evaluate the role of experience-dependent neuronal activity on barrel expansion in birth-enucleated rats by combining various anatomical methods and sensory deprivation paradigms. We show that barrels in birth-enucleated rats were already enlarged by the end of the first week of life and had levels of metabolic activity comparable to those in control rats at different ages. Dewhiskering after the postnatal period of barrel formation did not prevent barrel expansion in adult, birth-enucleated rats. Further, dark rearing and enucleation after barrel formation did not lead to expanded barrels in adult brains. Because incremental increases of somatosensory experience did not promote barrel expansion in birth-enucleated rats, we explored whether shifts of the developmental timing could better explain barrel expansion during the first week of life. Accordingly, birth-enucleated rats show earlier formation of barrels, accelerated growth of somatosensory thalamocortical afferents, and an earlier H4 deacetylation. Interestingly, when H4 deacetylation was prevented with a histone deacetylases inhibitor (valproic acid), barrel specification timing returned to normal and barrel expansion did not occur. Thus, we provide evidence supporting that shifts in developmental timing modulated through epigenetic mechanisms, and not increased levels of experience dependent neuronal activity, promote barrel expansion in the primary somatosensory cortex of rats enucleated at birth.

## Introduction

In mammals, prenatal and early postnatal eye damage leads to a large-scale reorganization of the brain that has been thoroughly documented in adults. This reorganization has been best characterized in the cerebral cortex, where the primary auditory and somatosensory areas expand and former visual neurons become activated by auditory and somatosensory stimuli [1]–[3]. Although the morphological, physiological and psychophysical consequences of cortical cross-modal plasticity in the brain of blind subjects have been extensively documented [4]–[6], the mechanisms underlying this massive plastic response remain, for the most part, unknown.

The primary somatosensory cortex (S1) of developing rodents contains a body map formed by modules of cell columns called barrels. This model system is often used to study mechanisms underlying cortical plasticity. Each barrel represents collections of mechanosensory receptors located beneath the body surface [7]. Previous work has shown that adult rodents enucleated at birth display expanded barrels [8], [9]. This expansion is thought to be promoted by increased use of whiskers following birth enucleation [9]. Presumably, this would result in incremental increases in levels of experience-dependent neuronal activity along the somatosensory pathway during postnatal development [8]–[10]. Contrary to this idea, previous work conducted in models of somatosensory intramodal plasticity showed that interfering with peripheral mechanosensory receptor function or knocking out NMDA receptor expression in S1 leads to overgrown thalamocortical somatosensory axons [11]–[13]. Furthermore, forelimb amputation in rat fetuses leads to a postnatal expansion of the cortical representation of the hind limb only if the amputation is carried out before embryonic day 17 [14]. Taken together, these observations suggest that cortical reorganization may proceed in the absence of incremental increases in experience-dependent neuronal activity. Thus, a re-evaluation of the role of experience-dependent neuronal activity in cross-modal plasticity is one of the principal aims of the present work.

In addition to neuronal activity elicited by sensory experience, what other factors could lead to barrel expansion in rodents enucleated at birth? Variation among the timing of developmental events (i.e., heterochrony) is now a well-recognized source of developmental phenotypic plasticity [15], [16]. Exaggerated or peramorphic physical traits may appear as a result of ontogenic processes that start early, last longer, proceed faster or finish later [15]. It is therefore possible that expanded barrels in enucleated rodents could result from changes in the timing of barrel formation and growth during early postnatal development. Hence, the second principal aim of our work was to evaluate this possibility.

A molecular event that controls developmental timing across eukaryotes is chromatin remodeling [17], [18]. In the mammalian brain, delaying histone deacetylation retards oligodendrocyte differentiation [18] and blocking the ability to methylate DNA, by knocking out the DNA methyltransferase 1 (*Dnmt1*), accelerates astrocyte differentiation [17]. Thus, the epigenetic modulation of chromatin structure may in part mediate shifts of the timing of various developmental processes underlying S1 barrel formation and growth in birth enucleated rodents. Supporting this possibility, conditional *Dnmt1* gene deletion in mice also disrupts S1 formation, impairs thalamocortical long-term potentiation and changes the electrotonic properties of thalamocortical synapses [19]. Hence, in this study we also addressed whether chromatin remodeling may in part mediate, by shifting developmental timing, cross-modal barrel plasticity in rats enucleated at birth.

## Materials and Methods

### Animals

Experiments were carried out in neonate and adult male Wistar rats, except for those used to determine the timing of barrel formation in which females were also included. All animals were raised in our animal facilities. For each litter, the number of pups was adjusted to eight; half were kept as control and half as experimental. This measure diminishes the effects of litter size and differential maternal care on individual brain development [20]. All animals had free access to food and water and were maintained in temperature and light controlled (12 hours light/12 hours dark cycles) rooms. The litters whose pups were dark-reared had also free access to food and water and were kept in a dark room from birth to the age of sacrifice.

Bilateral enucleation [8], [10] and unilateral whisker cauterization were carried out following protocols described in previous publications [21], [22]. All surgical manipulations were performed under anesthesia by hypothermia. Enucleation was performed in newborn rats within the first 10 hours after birth in all groups except for those pups that were enucleated 168 h postpartum. Rats sacrificed at PD30 and PD60 were weaned at PD21. For inmunohistochemistry experiments, neonate and young rats were maintained with their mothers from birth until sacrifice at 48 h, 82 h and 168 h. Between five to twenty-eight animals were used for each experimental condition (for details on the size of each cohort please read the corresponding section below). All of the animal protocols followed the guidelines published in the National Institutes of Health Guide for the Care and Use of Laboratory Animals, and were reviewed and approved by the Ethical Committee for the Care and use of Laboratory Animals (Permit No. 80) at the Instituto de Investigaciones Biomédicas, Universidad Nacional Autónoma de México. All surgical procedures were performed under anesthesia by hypothermia or following the administration of pentobarbital, and all efforts were made to minimize suffering of animals.

### Estimating Average Barrel Surface in the S1 Whiskers’ Cortical Representation

Rats were transcardially perfused with a 0.15 M saline solution followed by 3% buffered paraformaldehyde. The brains were removed and the cortical mantles were dissected, flattened between two glass slides, dehydrated with a gradient of sucrose and frozen in 2-methylbutane. Tangential sections (40 µm) were then cut in a cryostat and stained for CyOx as described by Riddle et al., 1993 [23]. These sections were used to draw two dimensional maps of the S1 posteromedial barrel subfield (PMBSF; from now on referred as barrels) with the aid of a camera *lucida* and used to precisely estimate average barrel surface area, as defined by Riddle *et al*., 1992 [24]. Each barrel was then outlined manually in every section it appeared. Radial blood vessels and barrels that appeared in multiple sections were used to align sections’ reconstructions. The outlines were superimposed following a top-down sequence and the final trace was integrated by using the largest profiles. The resultant maps were scanned and digitized. To estimate average barrel surface in different animal groups, digital masks were created for each barrel and the resulting maps were thresholded and converted into binary images that were used to estimate the surface of each of the 36 barrels that constitute the PMBSF (for details see [24]). The average barrel surface area in the PMBSF was then determined for each hemisphere in every animal evaluated. Average barrel size per group was estimated by averaging these last values. The procedures described were all performed using a computer-assisted image analysis system (Scion Image; ScionCorp, Beta 4.0.2). Average barrel size is a sensitive parameter to estimate overall PMBSF growth [24]. In our experimental series there were no changes in barrel number or geometry. Average barrel surface of experimental groups was compared against their age-matched control counterparts with a one-tailed unpaired Student’s *t* test at PD10 and PD60. Each experimental group consisted of eight animals with bilateral hemisphere analysis. In all statistical analyses, alpha level was set at 0.05 and p values are stated as *p≤0.05, **p≤0.01, and ***p≤0.001. All values are presented as mean ± SEM (Standard Error of the Mean).

### Estimating Cytochrome Oxidase Activity in Trigeminal Ganglion Neurons and PMBSF Barrels

Relative levels of CyOx activity were measured in longitudinal sections (40 µm) of trigeminal ganglia and tangential sections (40 µm) of the PMBSF of both control and birth-enucleated rats by using computer-assisted imaging densitometry (for further details see [23]). The analyses were performed blindly to avoid observer’s bias and three different individuals participated in the process of data collection. The values of gain, contrast and brightness were kept the same across sessions of image acquisition. Finally, bright and dark images were used to correct uneven illumination.

In the case of trigeminal ganglia, rats were euthanized with an overdose of sodium pentobarbital, the brain was removed and the ganglia were dissected. Each ganglion was embedded in a plastic capsule filled with OCT compound, frozen, cut in a cryostat and stained for CyOx (For further details see [25], [26]). In the case of barrels, rats were transcardially perfused with a 0.15 M saline solution followed by 4% buffered paraformaldehyde. The brains were removed and the cortical mantles were dissected, flattened between two glass slides, and frozen in 2-methylbutane. Sections were then cut in a cryostat and stained for CyOx. Because variations in CyOx activity might result from developmental changes in enzyme availability, we determined densitometrically the saturation point of the histochemical reaction for each of the regions analyzed at different ages. The saturation point was reached after 2 hours of incubation (37°C) for trigeminal ganglion sections and 8–10 hours of incubation (34°C) for brain slices irrespective of age and animal manipulation. Based upon these results, we decided to stop the histochemical reaction after one hour for trigeminal ganglion sections and after 5 hours for brain slices. Stained sections containing the PMBSF were captured (CoolPix Nikon 2272×2272 dpi) and digitized. Each barrel was outlined manually in every section it appeared. Within this outline, the average light transmittance per barrel was estimated using Scion Image. The unit of the densitometric values was arbitrary and provided automatically by the program. However, they are determined based upon a standard gray scale provided by Scion Image; the gray scale was the same for all of the measurements gathered. This last operation partly corrected for non-linearity of the distribution of the data set. The procedure just described was used to estimate the average light transmittance for each of the 36 barrels that form the PMBSF per hemisphere. Densitometric values per hemisphere were then calculated by summing up the average values of the 36 barrels divided by their total number. Finally, average barrel density of CyOx activity per animal group represents the mean density of all of the hemispheres analyzed. To correct for intrinsic variations of the staining, the density was estimated as a percent difference relative to the underlying corpus callosum. This procedure also corrects for non-linearity of the staining, and thus of light transmittance. Images of ganglion neurons were captured, digitized, processed and analyzed exactly as described for barrels. To correct for intrinsic variations of the staining, the density of the reaction product was estimated as a percent difference relative to the trigeminal nerve distal stalk. Each experimental group consisted of eight animals. One tailed unpaired Student’s *t* tests were performed.

### Estimating the Uptake of ^3^H-2 Deoxyglucose in the Cortical Representation of Vibrissae

Tritiated deoxyglucose (2DG; 3 µCi/gram of body weight; American Radiolabeled Chemical, St. Louis, MO, USA) was administered to both control and enucleated rats (for details see [23]). All of the experiments were conducted at 8pm to reduce circadian effects on brain metabolism and to match the natural phase of rat activity. After the administration of the 2DG the animals were returned to their cages for 45 minutes. Rats were then euthanized by an overdose of pentobarbital and decapitated. The brains were rapidly removed, hemissected, flattened, frozen and stored at −70°C until used. Serial brain tangential sections (20 µm) were cut in a cryostat, thaw mounted onto gelatin–coated coverslips and quickly dried on a hot plate. The coverslips were overlaid with tritium sensitive hyperfilm sheets (Amersham, Arlington Heights, IL, USA) and the films were exposed for three weeks at 4°C. After developing the films the sections were stained for CyOx. Images of the sections containing the PMBSF were captured and digitized. Next, the autoradiograms were aligned over the corresponding sections through fiducial marks present in both. Then, barrel profiles were manually outlined on the autoradiograms based upon the underlying barrels stained with CyOx. After removing CyOx stained reference images, the average light transmittance per barrel within such outlines was estimated in the autoradiograms. The rest of the procedure used to measure 2DG uptake, through densitometric means, followed the guidelines provided in the preceding section used to evaluating CyOx activity. Each experimental group consisted of four animals. One-tailed unpaired Student’s *t* tests were performed to carry out comparisons within age groups.

### Timing Barrel Specification

Control and birth-enucleated pups were deeply anesthetized on ice and decapitated between PD0 to PD5. It is worth to emphasize that the timing of birth was strictly monitored and precisely determined for each pup, and individual postnatal development carefully timed until their sacrifice. The brains were obtained and the cortical mantles dissected and flattened between two glass-slides separated by one millimeter. Cortical samples were then frozen and stored at −75°C until used. Tangential sections (50 µm) of the cortical mantle were obtained and stained for CyOx. We timed precisely the beginning of barrel formation at 80, 82, 84, 88, and 92 hours postpartum. Because the first barrels were more reliably observed at 82 hours postpartum (i.e., PD3/10 h) in some individuals of both rat groups, we decided to search for barrels at this age in a larger cohort of control and enucleated rats (the timing of birth and development of these groups was also strictly followed). The number of animals of both groups that showed barrels at this age was then recorded and compared. Each experimental group consisted of twenty-two to twenty-eight animals. The percentage of pups showing barrels at 82 and 92 h for control and birth-enucleated rats was compared with a two-tailed Fisher’s exact test for each age.

### Inferring the Rate of Growth and Size of Somatosensory Thalamocortical Afferents

Thalamocortical afferents were traced following injections of 1′-dioctadecyl-3,3,3″ tetramethylindocarbocyanine (DiI) in the ventral basal complex of the thalamus following the protocols provided in [11], [27], [28]. Briefly, control and birth-enucleated pups were anesthetized and perfused through the heart with saline solution followed by 10% buffered formalin at the age of 168 h. Following the perfusion, the animals were decapitated and their heads placed into jars containing the same fixative at room temperature. After a week, the heads were mounted on a stereotaxic frame and 1 µl of 1,1′-dioctadecyl-3,3,3″ tetramethylindocarbocyanine (DiI; 0.25% in ethanol; Molecular Probes, Eugene, OR, USA) was injected into the ventrobasal complex of the thalamus [27]. The heads were placed into light protected jars containing buffered paraformaldehyde and kept at room temperature for a month. After this time coronal sections (100 µm) through S1 were obtained in a vibrotome, mounted on gelatin-coated slides and coverslipped with anti-fading mounting medium (Dako, Carpinteria, CA, USA). PMBSF barrels were then identified in the slices of control and enucleated rats at equivalent anterior-posterior levels. Individual axonal arbors inside PMBSF barrels were drawn under fluorescent light with the aid of a camera *lucida*. The total length for each axon was measured in the digitized images according to the protocol described by [11]. Areal extent of somatosensory thalamocortical axons was estimated by outlining the tips of each afferent from two-dimensional reconstructions [28]. Each experimental group consisted of eight animals. Axonal area and length were compared between control and birth-enucleated rats at 168 h by a one tailed unpaired Student’s *t* test.

### Quantifying Nuclear H4 Acetylation in S1 Layer IV Cortical Neurons

Control and birth-enucleated rats of 48 h, 82 h and 168 h of life were anesthetized with pentobarbital and perfused with saline followed by 3.7% paraformaldehyde in phosphate buffer saline (PBS; pH 7.4, 0.1 M); perfusion lasted 25 minutes at room temperature. Whole brains were removed and postfixed overnight at 4°C and subsequently embedded in paraffin. Serial coronal sections (15 µm) were obtained using a microtome, mounted, and deparaffinized. Antigen retrieval was performed with citrate buffer (BioSBInc) during 90 min at 65°C. Sections were sequentially treated with 0.5% Triton X-100 in PBS for 60 minutes, 0.1 M glycine in PBS containing 1% bovine serum albumin (BSA) for 30 minutes and 0.01% Tween 20 in PBS for 5 minutes. Sections were incubated for 68 hours at 4°C with rabbit anti-acetylated histone H4 (H4ac; Chemicon) primary antibodies diluted (1∶500) in a solution containing 1% BSA, then washed with 0.01% Tween-20 in PBS, and finally incubated for 90 minutes with the corresponding Alexa 594 conjugated secondary antibodies with (Molecular Probes). By the end of this step, sections were washed in phosphate buffer (PB), counterstained with DAPI and mounted with DAKO anti-fading medium. Optical sections of S1 layer IV neuronal nuclei stained for H4ac were acquired using either a Carl Zeiss Pascal confocal microscope (82 h) or an Olympus B51 immunofluorescence microscope equipped with a disk scanning unit (48 h and 168 h) using a 100X oil objective.

Foci number counting is an established method for assessing histone mark dynamics ([29]–[33]). H4ac staining frequently displays a high variation in foci fluorescence intensity and background staining. Therefore, counting the precise number of individual foci per nucleus is the most reliable quantitative indicator of H4ac in immunostained brain tissue sections. The number of nuclear H4ac foci was estimated by using the particle analysis suite of ImageJ (Rasband, 1997–2001). Acquired images were digitally processed to generate binary masks that were obtained by visually adjusting the contrast until reaching the best image fit. Once defined, values of gain, contrast and brightness were kept the same for age group across imaging sessions. Next, the watershed algorithm was used to separate foci that touched each other with a maximum overlapping of 30%. Each experimental group consisted of eight to ten animals. H4ac foci number per neuronal nucleus obtained at every age evaluated was compared with the General Linear Model (GLM) using the Statistical Analyses System 9.2 (SAS Institute, Cary, NC, USA). Foci number variables were transformed with the Box-Cox procedure. The main effects and the interaction were included in the model.

### Modulating the Timing of Barrel Specification

Valproic acid (VPA, from Sigma) was administered to control and birth-enucleated pups through the motheŕs milk. For doing so, lactating dams were either injected intraperitoneally with valproic acid salt dissolved in PBS or with PBS alone, as a control, after parturition. VPA was administered at a dose of 600 mg/kg the first day and at 300 mg/kg from the second day until reaching 82 h or 168 h *postpartum*. Pups were perfused at the age of 82 h and 168 h. Barrels were then visualized and measured in tangential sections by using CyOx as described before. Each experimental group consisted of five to seven animals. The effects of VPA treatment on barrel size in control and enucleated rats were analyzed by using the GLM procedure, formerly mentioned, after a Log_10_ transformation of the data. Given that GLM analyses reached significant differences (*p*<0.05) among groups, a Tukey-Kramer *post-hoc* test was used to determine mean differences (*p*<0.05).

## Results

### Increased Sensory Experience is not Promoting Barrel Expansion in Birth-enucleated Rats

Whiskers that are longer than normal in enucleated rodents are assumed to reflect increased use of mechanosensory receptors [9]. Given that the cortical representations of the most posterior facial whiskers α, β, γ and δ consistently expand in rodents enucleated at birth [8]–[10], we precisely determined the length and growth rate of these whiskers in control and birth-enucleated rats at PD10, PD30 and PD60. We found no significant differences of whisker length between control and enucleated rats when compared at different ages (Fig. S1).

**Figure 1:**
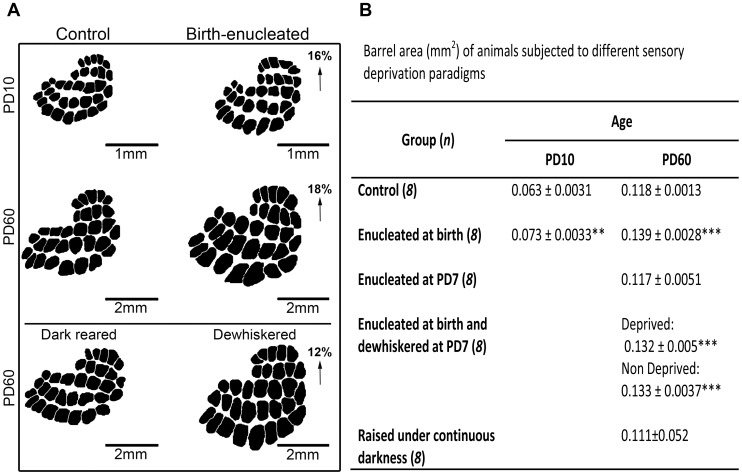
Experience dependent neuronal activity has a minor role in barrel expansion: Anatomical evidence. A) Representative two-dimension camera *lucida* reconstructions of the barrel field of animals subjected to different sensory deprivation paradigms. All reconstructions were derived from tangential sections stained for CyOx, similar to those shown in Fig. 2C. B) Table showing the mean barrel area in animals subjected to distinct sensory deprivation paradigms. One-tailed unpaired Student’s *t* tests: **p<0.01, ***p<0.001. Data are represented as the mean (+/− SEM).

Increased levels of experience-dependent neuronal activity along the trigeminal pathway during postnatal development are thought to promote barrel expansion in adult rats enucleated at birth [8]–[10]. We estimated and compared average barrel surface as previously defined [23] between control and birth-enucleated rats at PD10 and PD60 (Fig. 1A and B). Barrels were detected by staining for CyOx, since oxidative enzymes are known to precisely predict both the time of barrel formation and the spatial distribution of somatosensory thalamo-cortical afferents [23], [24], [34]. We found that the average surface of barrels located in the posteromedial barrel subfield (PMBSF) was 16% and 18% greater in rats enucleated at birth than in their sighted aged matched control counterparts at PD10 and PD60, respectively (Fig. 1A and B). Interestingly, barrels scaled up similarly in control (87%) and birth-enucleated (90%) rats between these ages. Together these results suggest that S1 expansion in rats enucleated at birth occurs primarily during the first ten days of life, and that increases in experience-dependent neuronal activity are not important for this process.

To further evaluate the role of experience dependent neuronal activity associated with S1 expansion, we also estimated average surface for PMBSF barrels in adult rats that had 1) their eyes removed at birth and were dewhiskered at PD10, 2) that were reared in darkness from birth and 3) that were enucleated at PD7. Enlarged barrels were found in the first group but had a normal surface in dark reared and PD7 enucleated rats (Fig. 1A and B). The fact that dewhiskering did not prevent barrel expansion in birth-enucleated rats and that dark-rearing and PD7 enucleation did not induce barrel expansion further supports the notion that the latter does not result from increases in experience dependent neuronal activity during postnatal development. In addition, these results suggest that it is the absence of the eyes, and not of visual experience, at early postnatal ages that conditions barrel expansion in birth-enucleated rats. The information used by the cortex to recognize the presence of the eyes may derive from the waves of spontaneous activity generated by the developing retina [35], [36]. Finally, since barrel expansion proceeds after birth enucleation but not following PD7 enucleation, our results support the idea that cross-modal plasticity occurs at a critical window of early postnatal development, as was previously suggested [37]–[39].

**Figure 2:**
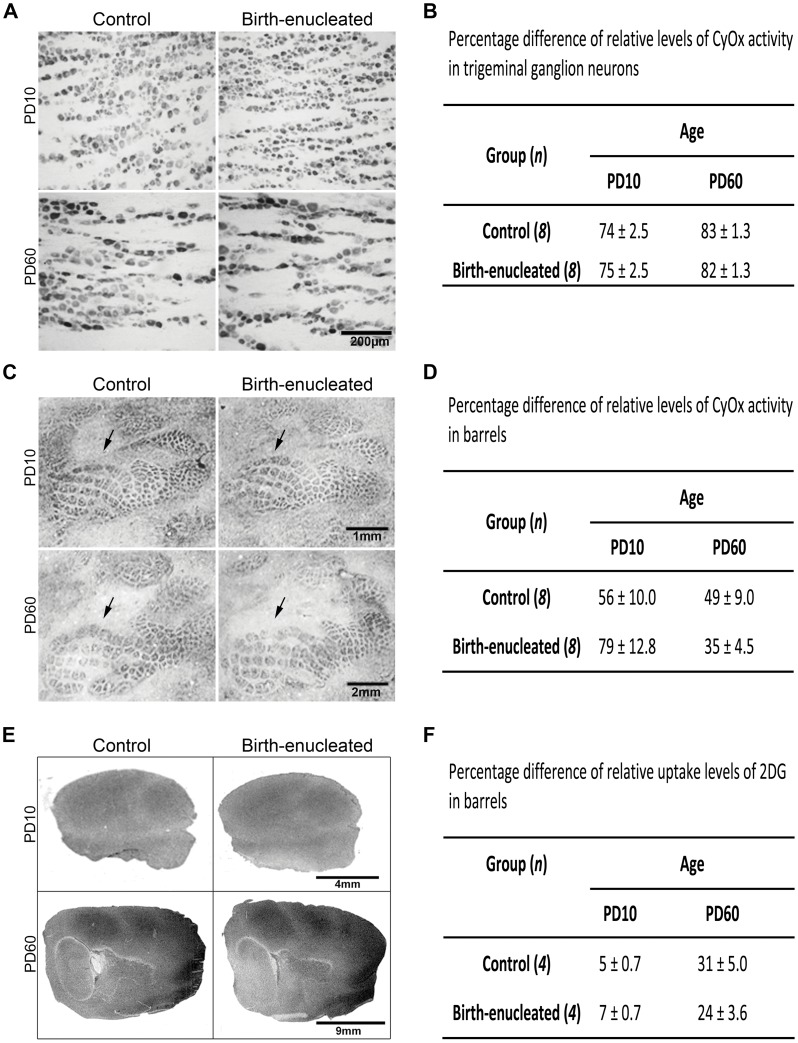
Trigeminal ganglion and cortical neuronal activity, as monitored through metabolic indices, did not differ between control and birth-enucleated rats at PD10 and PD60. A) Representative longitudinal sections through the middle third of the trigeminal ganglion visualized through CyOx staining. C) Representative tangential sections through S1 layer IV stained for CyOx; barrels are indicated by arrows. E) Representative ^3^H2-deoxyglucose (2DG) autoradiograms through S1 layer IV. B, D and F) Tables showing the mean percentage difference of the densitometric values obtained for each metabolic marker and condition. One tailed unpaired Student’s t tests: non-significant. Data are represented as the mean (+/− SEM).

Levels of neuronal activity can be more directly estimated by measuring cytochrome oxidase (CyOx) activity and H^3^ 2-deoxyglucose (2DG) uptake [23], [25], [26]. We estimated CyOx activity in the trigeminal ganglion (Fig. 2A and B) and CyOx and 2DG uptake in S1 of control and birth-enucleated rats at PD10 and PD60 (Fig. 2C–F). Densitometric analyses found CyOx activity in the trigeminal ganglion and CyOx and 2DG uptake in S1 to be similar in control and enucleated rats at both ages (Fig. 2). Hence, it is unlikely that expanded barrels in adult rats enucleated at birth result from incremental increases in experience-dependent neuronal activity along the trigeminal pathway during postnatal development.

### Birth-enucleated Rats Show an Earlier Formation of Barrels and an Accelerated Growth of Somatosensory Thalamocortical Afferents

**Figure 3:**
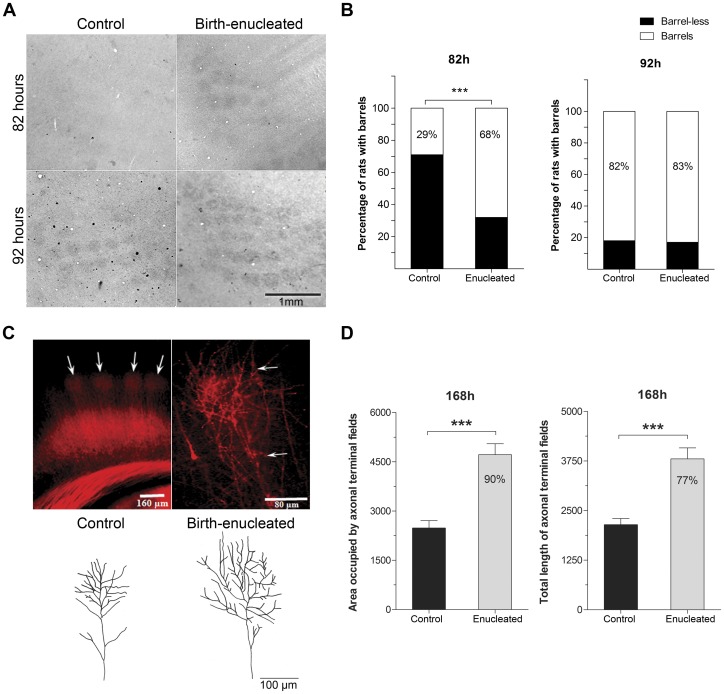
Earlier barrel specification and accelerated axonal growth in blinded rats. A) Representative tangential sections through S1 layer IV of control and birth-enucleated rat pups as visualized with CyOx histochemistry at 82 and 92 hours after birth. B) Percentage of animals with or without barrels at the evaluated ages. Two-tailed Fisher’s exact test: ***p<0.001. C) Representative coronal sections through the S1 barrel field (arrows) following DiI injections into the ventroposteromedial nucleus of the thalamus at 168 hours after birth (Right panel). The middle panel shows a higher magnification of the terminal fields of various DiI traced axons occupying the hollow of a barrel. Arrows indicate the approximate location of layer IV upper and lower limits. The left panel shows representative camera lucida drawings of axons of control and birth-enucleated pups. D) Graphs showing the mean axonal area and length of axonal terminal fields. One-tailed unpaired Student’s t tests: ***p<0.001. Data are represented as mean (+/− SEM).

Since increases in experience dependent neuronal activity did not seem to drive S1 expansion in rats enucleated at birth, we asked whether shifts in developmental timing could explain barrel expansion in these rats. Barrels were first noticeable after 82 hours (h) *postpartum* in 68% of the birth-enucleated rats. By the same age, only 29% of control rats displayed barrels (Fig. 3A and B) as determined by CyOx staining. These results were corroborated with Cresyl violet staining (Fig. S2). Hence, barrel specification occurs earlier during development following enucleation. Interestingly, by 92 hours this difference between groups essentially disappeared, suggesting that the endpoint of barrel field formation was not modified by enucleation.

The surface of individual barrels is specified and predicted by the terminal fields of the corresponding thalamocortical afferents projecting into them [34]. Hence, the larger-than-normal size of barrels at PD10 and the premature specification of barrels in birth-enucleated rats, along with the similarities in endpoint timing of barrel field formation between control and birth-enucleated rats suggest that somatosensory thalamocortical axons may be growing at a faster rate. Accordingly, these axons were longer and invaded a larger S1 surface as determined by using DiI tracing in rats enucleated at birth when compared with control animals at 168 h (Fig. 3C and D). These results confirm that the rate of axonal growth is enhanced in birth-enucleated rats during early stages of postnatal development.

### Chromatin Remodeling Occurs in S1 Layer IV Cortical Neurons of Birth-enucleated Rats

**Figure 4:**
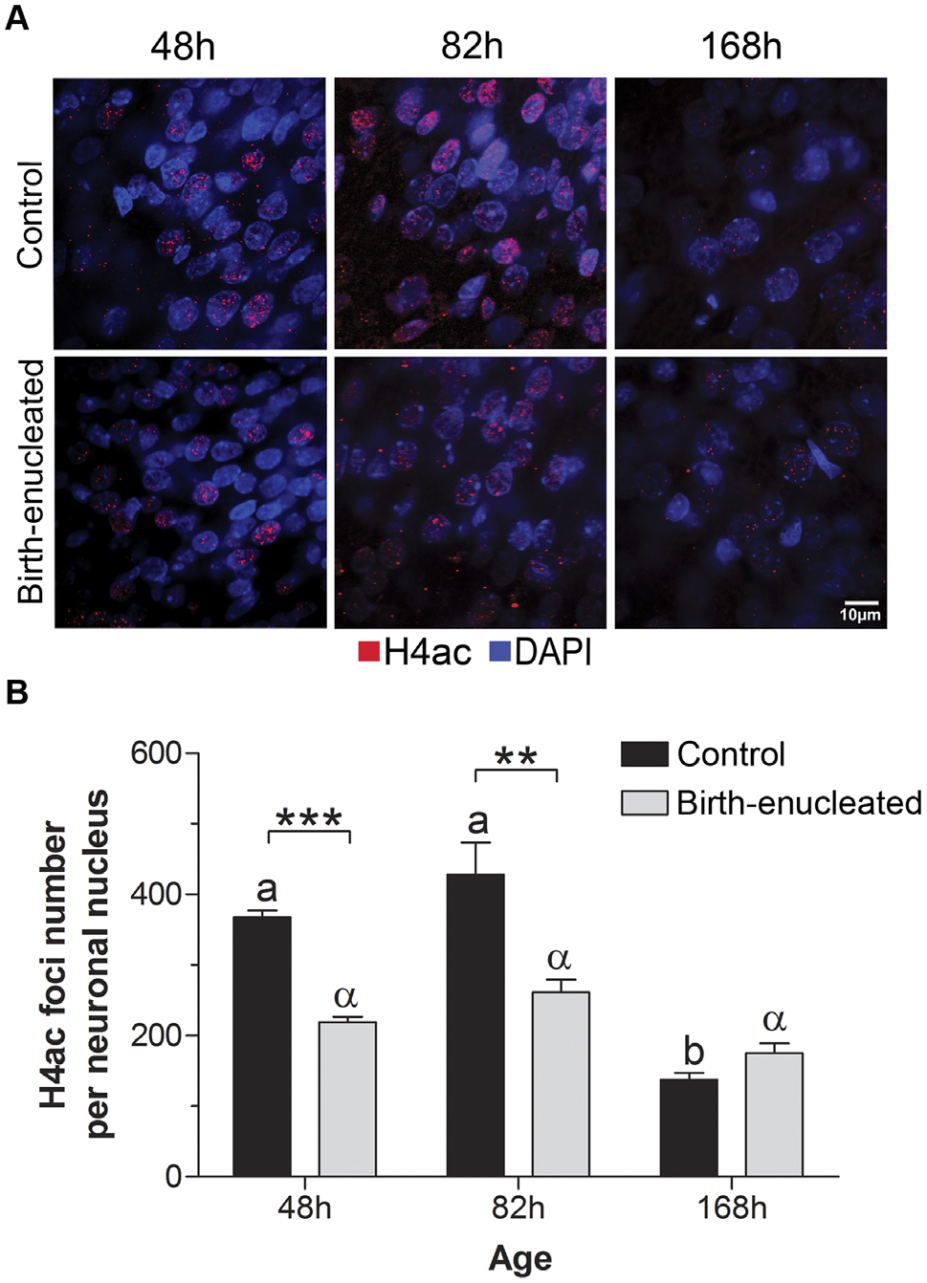
H4ac levels in S1 layer IV neurons decrease earlier during cortical development in blinded rats. A) Representative high magnification photomicrographs of a single Z-stack plane, showing coronal sections through layer IV of S1, immunostained for H4ac (red) and counterstained with DAPI (blue), in control and birth-enucleated animals at 48 h, 82 h and 168 h of life. B) H4ac positive foci number for each experimental condition. General Linear Model: **p<0.01, ***0.001. Within group comparisons across ages: bars with different literals are significant at *p*<0.05. Data are represented as the mean (+/− SEM).

Our results support the idea that shifts in developmental timing, but not increases in experience-dependent neuronal activity, facilitate the expansion of S1 in birth-enucleated rats during postnatal development. A molecular event that controls developmental timing across eukaryotes is chromatin remodeling [17], [18], [40]. One way to infer that chromatin remodeling has occurred is by monitoring shifts in the distribution and/or global amount of epigenetic marks extant in the chromatin; the addition or removal of methyl and acetyl groups to histones and/or DNA modifies chromatin configuration, and thus gene expression [41]–[44]. Global histone 4 acetylation (H4ac) decreases significantly in the brain between the end of embryonic life and the first days of postnatal development, as neuronal differentiation takes place [45]. Thus, we monitored the relative state of acetylation of histone 4 at 48, 82 and 168 hours of life in control and birth-enucleated rats though immunocytochemistry. We found that nuclear H4ac immunostained foci were reduced in S1 cortical layer IV neurons of birth-enucleated rats as compared with their control littermates at 48 h and 82 h of life. Importantly, this difference declined when rats of both groups reached 168 h of life (Fig. 4A and B). These data suggest that chromatin remodeling takes place coincidentally with the shift of the timing of barrel specification in birth-enucleated rats, and that the processes leading to barrel expansion are epigenetic in nature and likely triggered by eye loss.

#### A deacetylase inhibitor normalizes barrel specification timing and prevents barrel expansion in birth-enucleated rats

**Figure 5:**
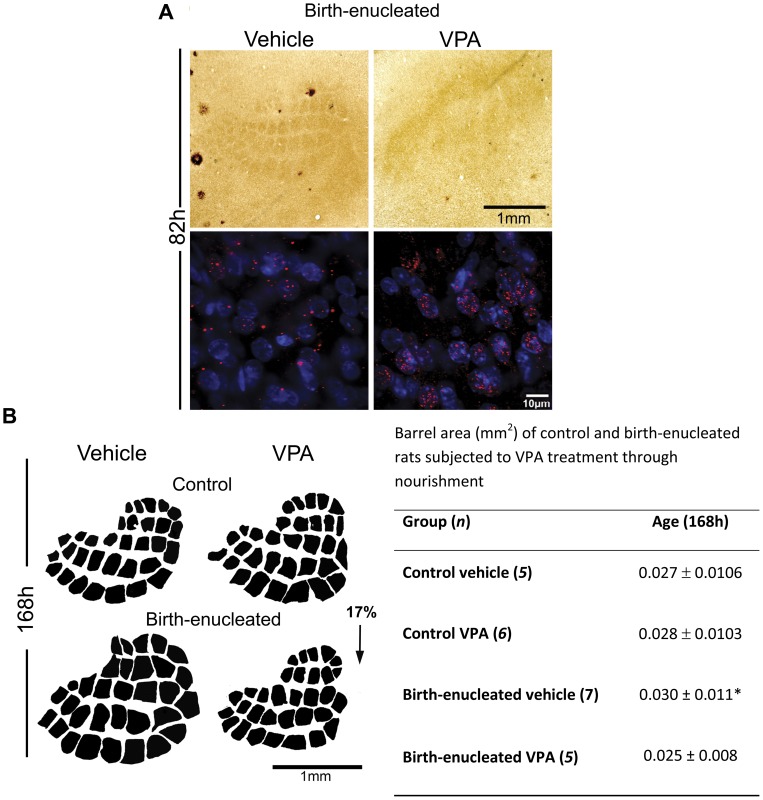
VPA treatment delays barrel formation, diminishes H4 deacetylation and prevents barrel expansion in birth-enucleated rats. A) Upper panel: Photomicrographs that illustrate tangential sections through S1 layer IV in 82 h old birth-enucleated pups treated or not with VPA as visualized with CyOx histochemistry; notice delayed barrel formation in the animals treated with VPA. Lower panel: Representative high magnification photomicrographs showing coronal sections through layer IV of S1, immunostained for H4ac (red) and counterstained with DAPI (blue), in 82 h old birth-enucleated pups treated or not with VPA. B) Upper panel: Barrel field two-dimension reconstructions obtained from 168 h old control and birth-enucleated animals treated or not with VPA. Lower panel: Table showing the mean barrel area in 168 h old control and birth-enucleated pups treated or not with VPA. General Linear Model followed by Tukey-Kramer *post-hoc* tests: *p*<0.05. Data are represented as the mean (+/− SEM).

The temporal coincidence of premature barrel specification and earlier layer IV neuron histone deacetylation suggest that both events might be causally linked. To address this possibility, we used the histone deacetylase inhibitor valproic acid (VPA), to acutely prevent chromatin remodeling before barrel expansion takes place. Control and birth-enucleated rat pups were fed with VPA through the mother’s milk [46]. Although this form of administration is more variable than injection, VPA feeding avoids subjecting pups to early stress associated with animal handling and mother separation, both conditions that have deleterious effects on brain postnatal development (e.g., [47]). In addition, it is now known that diverse elements contained in the eaten food are perfectly capable of modifying chromatin through epigenetic mechanisms [48]–[54]. Accordingly, 80% of birth-enucleated pups show barrels at the age of 82 h; by contrast, only 40% of the VPA fed, birth-enucleated rats show barrels at this age (Fig. 5A). Furthermore, VPA fed, birth-enucleated rats displayed higher levels of H4ac than normally fed, birth-enucleated rats at 82 h. Interestingly, VPA feeding prevented barrel expansion in birth-enucleated rats but had no effect on barrel size on VPA fed control rats at 168 hours of life (Fig. 5B). These results support the hypothesis that epigenetic regulation of developmental timing promotes barrel expansion in birth-enucleated rats during the first week of life.

## Discussion

Blind rodents display an expansion of S1 [9], [10]. Although previous work suggests that incremental increases in neuronal activity associated with enhanced somatosensory experience lead to S1 expansion in birth-enucleated rodents [9], [10], the present results weaken this notion. The observations that support our conclusions are: 1) The size and growth rate of whiskers in birth-enucleated rats are similar to those observed in control sighted rats; 2) The magnitude of barrel expansion was similar between young and adult rats enucleated at birth; 3) Unilateral dewhiskering at 168 h did not prevent barrel expansion in the deprived hemisphere nor did it produce barrel overgrowth in non-deprived hemispheres of birth-enucleated adult rats; 4) Neither enucleation after the period of barrel formation nor raising rats in complete darkness promoted barrel expansion; 5) Metabolic markers of neuronal activity (CyOx and 2DG) were comparable in the trigeminal ganglion and/or in non-expanded and expanded PMBSF barrels of control and birth-enucleated rats, respectively, at different ages; and 6) Barrel expansion in birth-enucleated rats took place before the onset of active whisking (PD12)[55]–[59], and before cortical-evoked activity [60]–[62].

Although our results challenge the classical view regarding the participation of evoked neuronal activity on cross-modal plasticity, our data agree with previous studies showing that both S1 specification and intramodal plasticity still proceed in the absence of experience-dependent neuronal activity at cortical [61], [63]–[65] or peripheral [66] levels. Furthermore, increased neuronal activity may favor growth cone collapse [67], [68], the production of axonal growth “stop” signals [69], the inhibition of axonal and dendritic filopodia movements [70], the decrease of the probability of axons and dendrites to grow new branches [71], the elimination of synapses [72] and substrate adhesiveness of axons [73]. Conversely, sensory deprivation leads to longer, and in some instances, more complex axons [11]–[13], [74], [75], increments of growth associated protein-43 availability [76], [77], and barrels that are larger than normal [78]. So, plasticity is more likely to occur under circumstances in which experience-dependent neuronal activity is not enhanced. Nonetheless, our results do not rule out the participation of spontaneous neuronal activity on barrel expansion in birth-enucleated rats. Indeed, a recent study showed that the mode and growth rate of somatosensory thalamocortical axons are intrinsically regulated based on the patterns of intracellular calcium transients [79]. These observations warrant future studies on the role of spontaneous activity in barrel expansion of birth-enucleated rodents.

If incremental increases in experience dependent neuronal activity during postnatal development do not explain barrel expansion in birth-enucleated rats, what is the cause? Shifting the timing of barrel specification and/or the rate of growth of its constituent cytological elements might well explain it. Accordingly, we showed that barrel specification occurs at least 10 hours earlier in birth-enucleated rats. The fact that thalamocortical axons are longer and distribute over a larger surface within barrels in birth-enucleated than in control rats at PD7 supports the view that they grow faster. Hence, both observations suggest that expanded barrels result from the combined effects of specifying barrels earlier and accelerating the growth rate of the incoming constituent axons. A more definitive demonstration of this hypothesis, however, required the experimental manipulation of the timing of barrel specification and/or of the growth rate of somatosensory thalamocortical axon prior barrel formation. We partly achieved this goal by delaying H4 deacetylation, and thus by epigenetically modulating chromatin remodeling, in S1 cortical neurons following VPA ingestion by birth-enucleated rat pups. Indeed, VPA fed, birth-enucleated pups had barrels specified at a normal age. These experimental conditions prevented barrel expansion at 168 h of life. Thus, our results clearly support that premature barrel specification is sufficient for developing enlarged barrels during the first week of life. Clarifying the role of a shifted somatosensory axonal growth rate on barrel expansion in birth-enucleated rats remains to be determined. In the meantime, our results support the notion that shifts in developmental timing modulated through epigenetic mechanisms, and not increased levels of sensory experience, promote barrel expansion in the primary somatosensory cortex of rats enucleated at birth. In addition, the enlarged barrels observed in adult rodents enucleated at birth result from the isometric growth of the perinatally enlarged axon terminals rather than from incremental increases in experience dependent neuronal activity along the trigeminal pathway as animals mature; CyOx activity and 2DG uptake were similar in trigeminal and/or S1 of control and birth-enucleated rats at PD10 or PD60 (see also [80] for evidence supporting S1 isometric growth in the normal developing rat brain).

It is worthwhile to mention the caveats that must be considered when interpreting our experimental results using VPA. First, although we showed that VPA delayed the process of nuclear H4 deacetylation in S1 layer IV neurons, VPA is likely also acting on thalamic and other neuronal and non-neuronal cell populations across the brain since it was provided through feeding. However, the fact that VPA prevented barrel expansion in birth-enucleated rats,without affecting barrel size in VPA-fed control rats support some degree of specificity of the VPA treatment. Furthermore, it is likely that VPA not only exerts pharmacological actions on histone/DNA deacetylating enzymes, it may also affect DNA methylation [81]. Although current results do not rule out this issue, preliminary work by our laboratory has shown that global DNA methylation is similar between control and birth-enucleated rats at 48, 82 and 168 hours of age (Fig. S3). In addition, VPA may increase the availability of GABA. Because GABA exerts excitatory actions at early postnatal developmental stages [82], [83] and the activation of developing neurons causes growth cone collapse [35], [67]–[69], VPA might be also preventing barrel expansion by increasing local excitability. Additionally, the maturation of local GABAergic transmission is critical to time, in a Otx-2 dependent manner, the onset of sensitive periods for cortical plasticity [84]–[87]. Hence, by increasing GABA availability, VPA could counterbalance the effect of enucleation and thus restore the timing of barrel specification. Lastly, VPA may be acting on the timing of barrel specification indirectly by modifying signaling cascades in which other substrates of histone deacetylases’ such as the androgen receptors, p53, MyoD, E2F1, STAT3, are involved [88]. Clearly, future research must address the specific contributions of each of these mechanistic possibilities.

A fundamental inference made based on our findings is that chromatin remodeling may be a central piece in the molecular machinery governing barrel expansion in birth-enucleated developing rodents. It is now accepted that the degree of chromatin compaction or relaxation controls gene expression [41], [42], [89]. The fact that nuclear H4 deacetylation occurs earlier in S1 cortical neurons in enucleated rats as compared to what is observed in control rats suggests that chromatin structure in S1 is being affected by the presence or absence of the eyes. The cross-modal regulation of chromatin remodeling at the cortical level could maintain genes involved in cortical parcellation such as ephrin-5 [90], [91] and fibroblast growth factor 8 [92] active for a longer time and/or decrease the expression of genes, such as Robo1 and Slit1 [79], that slowdown the rate of somatosensory axonal growth via spontaneous patterns of activity allowing barrels to expand. In addition, genes involved in regulating S1 availability of acetylcholine [93], serotonin [94], [95] and growth associated protein-43 [96], all previously shown to control barrel size, may also be affected by chromatin remodeling in S1 neurons associated with enucleation. Finally, because enucleation retards the onset of visual cortical plasticity by preventing Otx-2 from reaching cortical GABAergic interneurons (and hence their maturation) from the retina [87], the combined effects of enucleation on the visual and somatosensory cortex could facilitate barrel expansion. In this regard, even though interactions between sensory cortical areas likely influence cortical reorganization, He et al. (2012) have shown that crossmodal and intramodal plasticity following different classes of visual deprivation go on independent from each other and relay on distinct sensory requirements [97].

Lastly, our work addresses a number of issues related to the cellular mechanisms influencing the reorganization of cortical connectivity in birth-enucleated rats. Previous reports suggest that the reorganization of the cortex in enucleated rodents involves the sprouting of callosal connections without affecting thalamocortical projections [98], [99]. In contrast, we provide evidence that shows that somatosensory thalamocortical axons do in fact undergo plasticity. We therefore suggest that somatosensory thalamocortical axons are key to barrel expansion in enucleated rats.

Further, Tratchtenberg and Stryker (2001) suggest that the anatomical plasticity of intracortical connections precedes the plasticity of thalamocortical afferents during ocular dominance columns reorganization [100]. In the rodent S1, intracortical connections develop later [101] than thalamocortical afferents and have a sensitive period that ends later than that of the thalamocortical afferents [102]. In our work, thalamocortical axons have already reorganized in the barrel cortex of birth-enucleated rats by the age of PD10. Hence, the plasticity of somatosensory thalamocortical afferents precedes the reorganization of corticocortical connections in enucleated rats.

The observed expansion of S1 barrels in neonatally enucleated animals with unilateral whisker cauterization at PD7 may in part be explained by interhemispheric transfer of plasticity [103], [104] and/or plasticity of callosal/cortico-cortical connectivity [99]. Our experimental approach cannot clarify the contribution of these processes to the expansion of S1 in enucleated/unilaterally dewhiskered rats. Because of the difficulties of raising bilaterally cauterized rats, combining enucleation, unilateral dewhiskering and callosotomy would be necessary to address this issue in the future.

Developmental cortical reorganization is thought to result from the retention of transient connections that would otherwise be eliminated through Hebbian competition (although see [105]). The growth of thalamocortical afferents in the barrel cortex is highly precise and barrel neuropil is added, not eliminated, as development proceeds [12], [34], [58], [106]–[109]. It is thus likely that the expansion of barrels in enucleated rats results from the addition of neuronal processes, not from retaining transient ones. The increasing size of barrel surface with age in both groups of animals and the presence of larger barrels and of thalamocortical axons longer and wider at PD7 in enucleated rats support the predominance of constructive events during barrel plasticity in enucleated rats.

### Conclusions

Here we report that experience-dependent neuronal activity plays a minor role in developmental cross-modal plasticity. We also provide novel evidence that supports the notion that S1 expansion in enucleated neonatal rats is likely due to an earlier specification of S1 that can be in part modulated through epigenetic mechanisms. This notion is a departure from the classical view that sustains that all forms of neuronal plasticity are promoted by incremental increases in neuronal activity elicited by the interaction of an organism with its surrounding environment.

## Supporting Information

Figure S1 **The Length and growth rate of whiskers are similar between control and birth-enucleated rats at different ages.** A, B and C) Length of α, β, γ and δ whiskers at PD10, PD30 and PD60 in control and birth-enucleated rats. At PD60 the whisker stem contains a primary (older) and a secondary (still growing) whisker. D) Growth rate of α, β, γ and δ whiskers during the second cycle in control and birth-enucelated rats. Whisker trimming was performed at the age of PD40. One tailed unpaired Student’s t tests: non-significant. Data are represented as the mean (+/− SEM). Materials and Methods: Estimating whisker length. Rats were euthanized with pentobarbital and their whisker pads were dissected and immersed in 4% buffered paraformaldehyde for a week at 4°C. Whiskers were carefully dissected with the aid of a stereoscopic microscope (Nikon SMZ1500 C-DSD, Nikon, Tokyo, Japan) and placed in PB. Whiskers with incomplete follicles or broken tips were discarded from the study. PD10 whiskers were photographed (1024 dpi) against a dark background through a stereoscopic microscope equipped with a digital camera (Nikon Coolpix 995, Nikon, Tokyo, Japan). PD30 and PD60 whiskers were captured with a scanner HP scanjetIIc at a resolution of 1200 dpi (Hewlett-Packard, USA). Whisker images were used to measure their length (in millimeters) from the base of the follicle to the whisker’s tip using a computer-assisted image analysis system (Scion Image; ScionCorp, beta 4.0.2). Whiskers from five to eight animals were analyzed. Average length was estimated per animal group and a one tailed t-Student test with p<0.05 was carried out. Whisker growth rate and growth cycle duration were estimated by measuring weekly their length increment during the second cycle of the whiskers growth (PD40). Estimating whisker growth rate. Whiskers α, β, γ and δ were trimmed at PD40 and the process of re-growth was monitored. The duration of whisker growth cycle was estimated between the first time point of the curve and the time point where it reached the asymptote. Whisker growth rate was obtained by determining the slope of the growth curve. Rats were under halothane anesthesia during measurements. Whiskers were then introduced into capillary glass tubes that were pressed against the skin in the mystacial pad. The external wall of the capillary was marked where the tip of whisker was observed. This mark was registered on millimetric paper sheets during seven weeks. These sheets were scanned and the distance between consecutive reference dots was measured with the aid of Scion Image. Average whisker growth rate was estimated per animal group and a one tailed t-Student test with p<0.05 was carried out. Reference: Ibrahim L, Wright EA (1975) The growth of rats and mice vibrissae under normal and some abnormal conditions. J Embryol Exp Morphol 33∶ 831–844.
(TIF)

Figure S2 **Barrels specify earlier in birth-enucleated rats.** Representative cresyl violet-stained, S1 tangential sections of control (A) and enucleated (B and C) rats at 82 hours of age. The outlines in C encircle the profiles of PMBSF barrels (D and E rows; lower left). Six out of nine birth enucleated pups, but none of the control pups, displayed barrel profiles as those illustrated in B. Dissected brains from rat pups used for these experiments were fixated with buffered paraformadehyde (4%) and the cortical mantles peeled off, cut (200 µm) with a vibratome, mounted onto gelatin-coated slides, stained with cresyl violet and coverslipped with cytoseal. Scale Bar = 500 µm.
(TIF)

Figure S3 **DNA 5 Methylcitosine (5meC) levels display no differences between control and birth-enucleated S1 cortical tissue.** Bar graph showing the percentage of global 5meC in sighted and blinded rats at 48 h, 82 h and 168 h obtained by HPLC. 2-way ANOVA: non-significant between groups across ages; **p<0.01 across ages. Data are represented as the mean (+/− SEM). Materials and Methods: Quantifying 5- methyl-cytosine (5meC) in genomic DNA. Total DNA and RNA from S1 samples of sighted and blinded rats were isolated (48 h n = 15, 82 h n = 24 and 168 h n = 12) using the All Prep DNA/RNA/Protein Kit (Qiagen). Briefly, brain tissue was homogenized with lysis buffer using a Tissue lyser (Qiagen). The lysates were purified using the DNA and RNA columns in the Allprep Kit (Qiagen) as suggested by manufacturer’s instructions. The concentration of the purified sample was measured with the Nanodrop 1000 Spectrophotometer. DNA samples were hydrolyzed with a solution containing 70% perchloric acid for one hour at 100°C. At the end, the reaction was neutralizedwith 10 N sodium hydroxide and filtered through a nylon filter cartridge (4 mm in diameter and 0.2 µm pore size). The samples were kept at 4°C until used. 5meC chromatographic separation (YMC-Pack ODS column; 5 µm, 250×4.6 mm) and quantification (UV-VIS detector at a wavelength of 280 nm) was performed by reverse phase liquid chromatography (Waters).The flow rate was 1.0 mL/min and a linear gradient elution of 5 mM ammonium acetate buffer added with glacial acetic acid (pH 3.5) and methanol was generated. 5meC concentration was determined by using cytosine and guanine (0.07 mM), adenine and thymine (0.05 mM); uracil (0.04 mM) and 5-meC (0.003 mM) as external standards. The percentage of cytosine methylation was estimated according to the method reported in Corvetta *et al.* 1991. Since 5meC levels are expressed as percentages, an Arcsin transformation was used to allow for normal distribution and homogeneity of variances, among evaluated groups of animals, and a 2-way ANOVA analysis was performed. Reference: Corvetta A, Della Bitta R, Luchetti MM, Pomponio G (1991) 5-Methylcytosine content of DNA in blood, synovial mononuclear cells and synovial tissue from patients affected by autoimmune rheumatic diseases. J Chromatogr 566∶ 481–491.
(TIF)
