# Supplementary figures and images for: Shifts in Developmental Timing, and Not Increased Levels of Experience-Dependent Neuronal Activity, Promote Barrel Expansion in the Primary Somatosensory Cortex of Rats Enucleated at Birth
Source: PLoS One. 2013 Jan 25;8(1):e54940. doi: 10.1371/journal.pone.0054940 (PMC3556040; doi:10.1371/journal.pone.0054940)

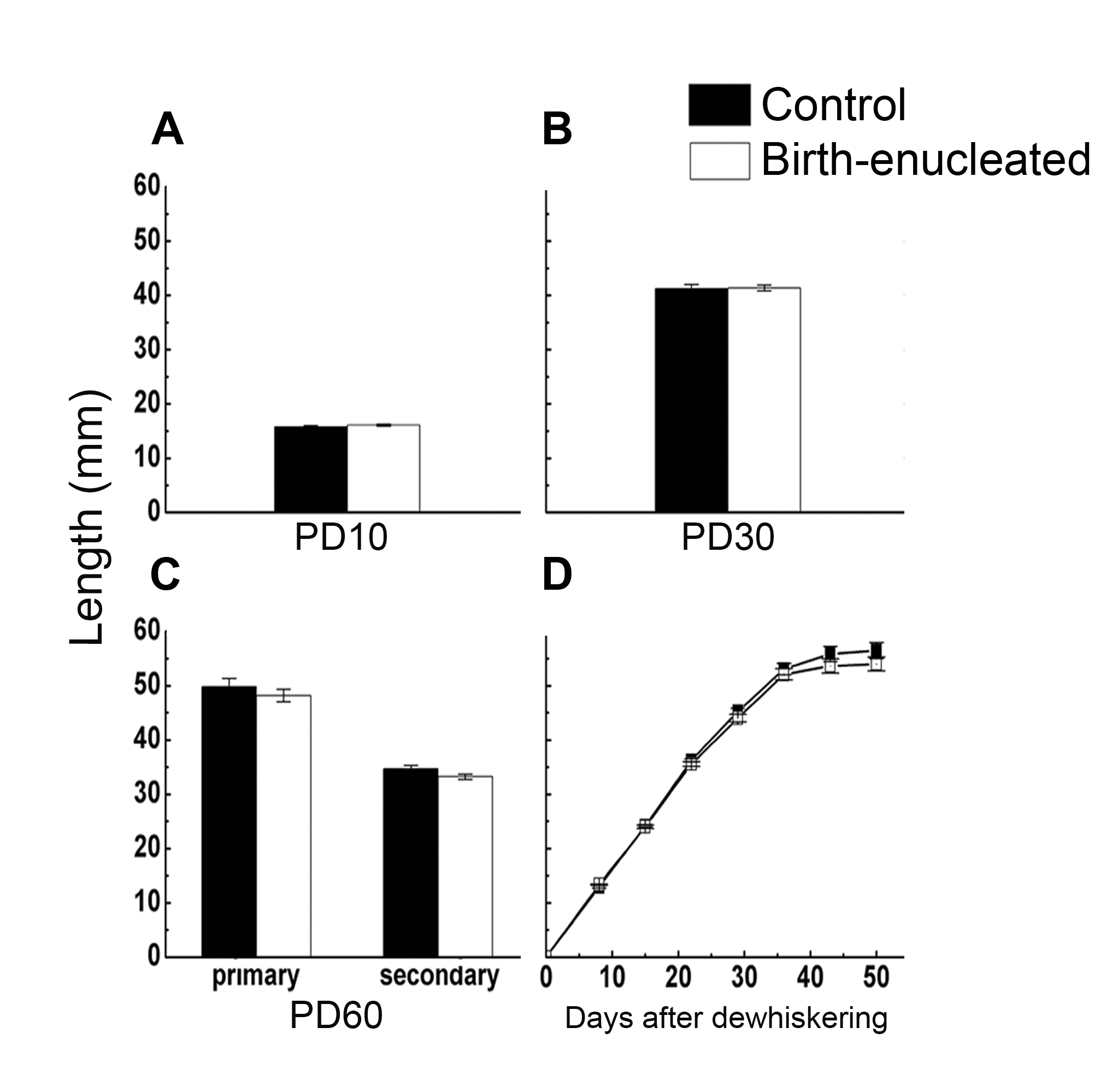

Supplement: Figure S1 — The Length and growth rate of whiskers are similar between control and birth-enucleated rats at different ages. A, B and C) Length of α, β, γ and δ whiskers at PD10, PD30 and PD60 in control and birth-enucleated rats. At PD60 the whisker stem contains a primary (older) and a secondary (still growing) whisker. D) Growth rate of α, β, γ and δ whiskers during the second cycle in control and birth-enucelated rats. Whisker trimming was performed at the age of PD40. One tailed unpaired Student’s t tests: non-significant. Data are represented as the mean (+/− SEM). Materials and Methods: Estimating whisker length. Rats were euthanized with pentobarbital and their whisker pads were dissected and immersed in 4% buffered paraformaldehyde for a week at 4°C. Whiskers were carefully dissected with the aid of a stereoscopic microscope (Nikon SMZ1500 C-DSD, Nikon, Tokyo, Japan) and placed in PB. Whiskers with incomplete follicles or broken tips were discarded from the study. PD10 whiskers were photographed (1024 dpi) against a dark background through a stereoscopic microscope equipped with a digital camera (Nikon Coolpix 995, Nikon, Tokyo, Japan). PD30 and PD60 whiskers were captured with a scanner HP scanjetIIc at a resolution of 1200 dpi (Hewlett-Packard, USA). Whisker images were used to measure their length (in millimeters) from the base of the follicle to the whisker’s tip using a computer-assisted image analysis system (Scion Image; ScionCorp, beta 4.0.2). Whiskers from five to eight animals were analyzed. Average length was estimated per animal group and a one tailed t-Student test with p<0.05 was carried out. Whisker growth rate and growth cycle duration were estimated by measuring weekly their length increment during the second cycle of the whiskers growth (PD40). Estimating whisker growth rate. Whiskers α, β, γ and δ were trimmed at PD40 and the process of re-growth was monitored. The duration of whisker growth cycle was estimated between the first time point of the curv [file pone.0054940.s001.tif]

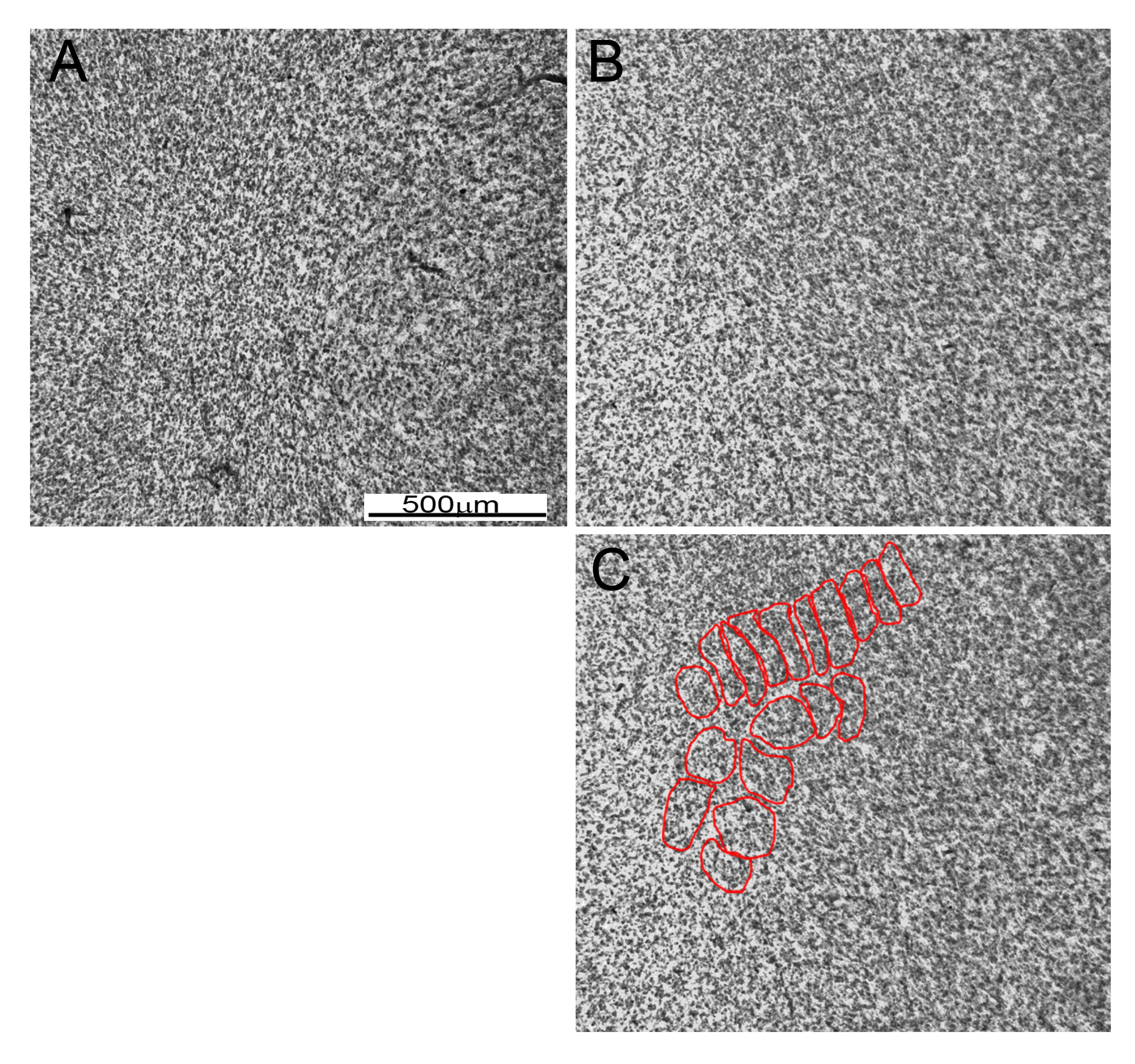

Supplement: Figure S2 — Barrels specify earlier in birth-enucleated rats. Representative cresyl violet-stained, S1 tangential sections of control (A) and enucleated (B and C) rats at 82 hours of age. The outlines in C encircle the profiles of PMBSF barrels (D and E rows; lower left). Six out of nine birth enucleated pups, but none of the control pups, displayed barrel profiles as those illustrated in B. Dissected brains from rat pups used for these experiments were fixated with buffered paraformadehyde (4%) and the cortical mantles peeled off, cut (200 µm) with a vibratome, mounted onto gelatin-coated slides, stained with cresyl violet and coverslipped with cytoseal. Scale Bar = 500 µm. (TIF) [file pone.0054940.s002.tif]

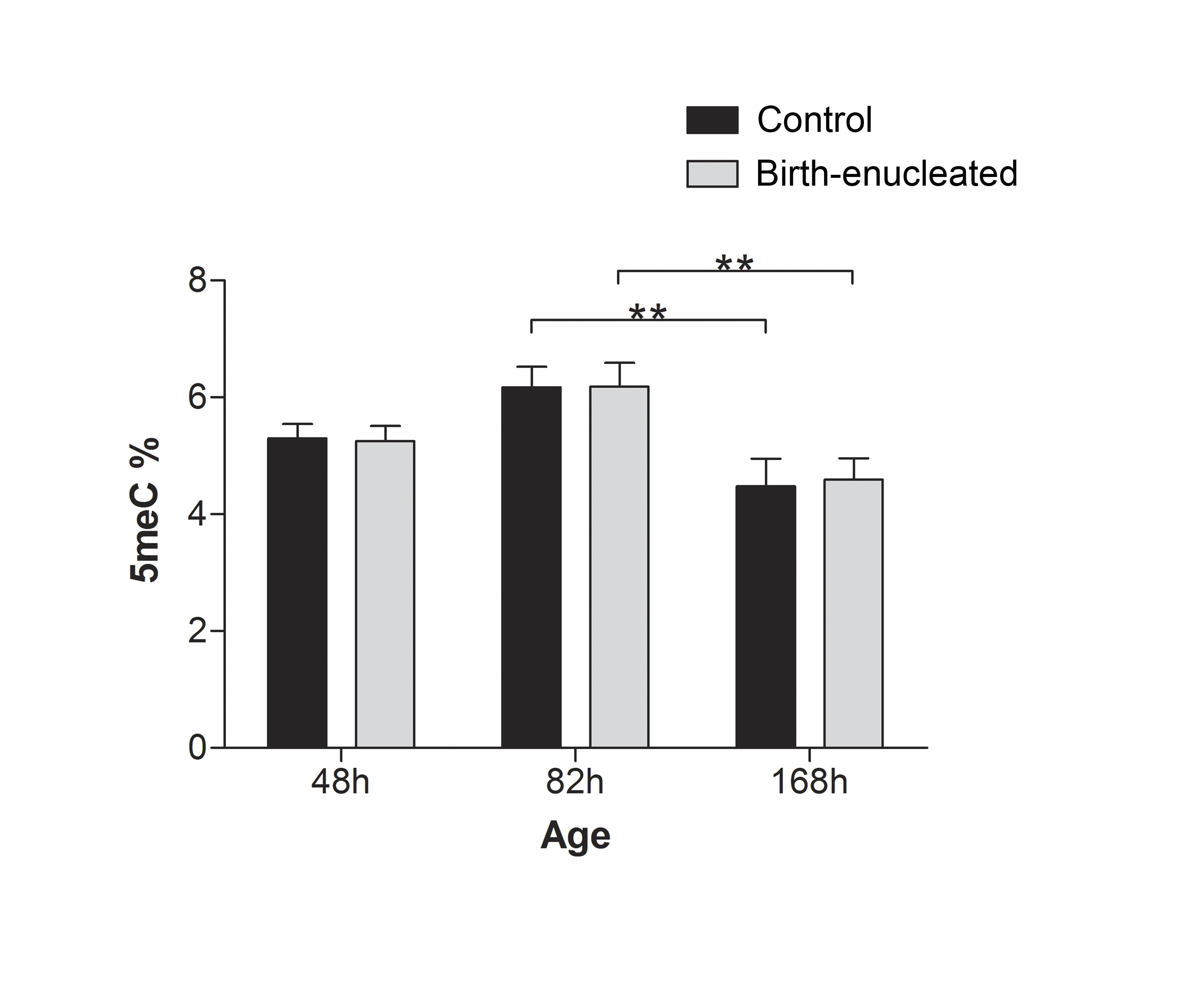

Supplement: Figure S3 — DNA 5 Methylcitosine (5meC) levels display no differences between control and birth-enucleated S1 cortical tissue. Bar graph showing the percentage of global 5meC in sighted and blinded rats at 48 h, 82 h and 168 h obtained by HPLC. 2-way ANOVA: non-significant between groups across ages; **p<0.01 across ages. Data are represented as the mean (+/− SEM). Materials and Methods: Quantifying 5- methyl-cytosine (5meC) in genomic DNA. Total DNA and RNA from S1 samples of sighted and blinded rats were isolated (48 h n = 15, 82 h n = 24 and 168 h n = 12) using the All Prep DNA/RNA/Protein Kit (Qiagen). Briefly, brain tissue was homogenized with lysis buffer using a Tissue lyser (Qiagen). The lysates were purified using the DNA and RNA columns in the Allprep Kit (Qiagen) as suggested by manufacturer’s instructions. The concentration of the purified sample was measured with the Nanodrop 1000 Spectrophotometer. DNA samples were hydrolyzed with a solution containing 70% perchloric acid for one hour at 100°C. At the end, the reaction was neutralizedwith 10 N sodium hydroxide and filtered through a nylon filter cartridge (4 mm in diameter and 0.2 µm pore size). The samples were kept at 4°C until used. 5meC chromatographic separation (YMC-Pack ODS column; 5 µm, 250×4.6 mm) and quantification (UV-VIS detector at a wavelength of 280 nm) was performed by reverse phase liquid chromatography (Waters).The flow rate was 1.0 mL/min and a linear gradient elution of 5 mM ammonium acetate buffer added with glacial acetic acid (pH 3.5) and methanol was generated. 5meC concentration was determined by using cytosine and guanine (0.07 mM), adenine and thymine (0.05 mM); uracil (0.04 mM) and 5-meC (0.003 mM) as external standards. The percentage of cytosine methylation was estimated according to the method reported in Corvetta et al. 1991. Since 5meC levels are expressed as percentages, an Arcsin transformation was used to allow for normal distribution and homogeneity of variances, among evaluate [file pone.0054940.s003.tif]
